# The chaperone domain BRICHOS prevents CNS toxicity of amyloid-β peptide in *Drosophila melanogaster*

**DOI:** 10.1242/dmm.014787

**Published:** 2014-03-28

**Authors:** Erik Hermansson, Sebastian Schultz, Damian Crowther, Sara Linse, Bengt Winblad, Gunilla Westermark, Jan Johansson, Jenny Presto

**Affiliations:** 1 KI Alzheimer Disease Research Centre, NVS Department, Karolinska Institutet, Novum, 5th Floor, 141 86 Stockholm, Sweden.; 2 Department of Biochemistry, Institute for Cancer Research, Oslo University Hospital – The Norwegian Radium Hospital, 0379 Oslo, Norway.; 3 Department of Genetics, University of Cambridge, Downing Street, Cambridge, CB2 3EH, UK.; 4 Department of Biochemistry and Structural Biology, Lund University, PO Box 124, 221 00 Lund, Sweden.; 5 Department of Medical Cell Biology, Uppsala University, 751 23 Uppsala, Sweden.; 6 Department of Anatomy, Physiology and Biochemistry, Swedish University of Agricultural Sciences, The Biomedical Centre, 751 23 Uppsala, Sweden.; 7 Institute of Mathematics and Natural Sciences, Tallinn University, Narva mnt 25, 101 20 Tallinn, Estonia.

**Keywords:** Amyloid, Alzheimer’s disease, Protein misfolding, Chaperone

## Abstract

Aggregation of the amyloid-β peptide (Aβ) into toxic oligomers and amyloid fibrils is linked to the development of Alzheimer’s disease (AD). Mutations of the BRICHOS chaperone domain are associated with amyloid disease and recent *in vitro* data show that BRICHOS efficiently delays Aβ42 oligomerization and fibril formation. We have generated transgenic *Drosophila melanogaster* flies that express the Aβ42 peptide and the BRICHOS domain in the central nervous system (CNS). Co-expression of Aβ42 and BRICHOS resulted in delayed Aβ42 aggregation and dramatic improvements of both lifespan and locomotor function compared with flies expressing Aβ42 alone. Moreover, BRICHOS increased the ratio of soluble:insoluble Aβ42 and bound to deposits of Aβ42 in the fly brain. Our results show that the BRICHOS domain efficiently reduces the neurotoxic effects of Aβ42, although significant Aβ42 aggregation is taking place. We propose that BRICHOS-based approaches should be explored with an aim towards the future prevention and treatment of AD.

## INTRODUCTION

Protein misfolding is the underlying cause of at least 30 amyloid diseases, including prevalent disorders such as Alzheimer’s disease (AD), Parkinson’s disease and type 2 diabetes (Sipe et al., 2012). These diseases are associated with a specific form of misfolding, where proteins aggregate into amyloid fibrils with β-strands running perpendicular to the fibril axis (Maji et al., 2009). Amyloid-β peptide (Aβ) was first isolated from meningeal vessels of individuals with AD, and then recognized as the main component of the senile plaques observed in AD brain tissue (Glenner and Wong, 1984; Masters et al., 1985). Aβ is derived from the transmembrane (TM) region of amyloid precursor protein (APP). Amyloidogenic Aβ, mainly consisting of 42 residues, is released by β- and γ-secretase (Haass, 2004). The amyloid cascade hypothesis posits that processing of APP produces Aβ, which then aggregates and forms fibrils, giving rise to synaptotoxicity and eventually causing dementia (Hardy and Selkoe, 2002). Many studies indicate that pre-fibrillar intermediates, including small soluble oligomers, are most potent in causing neuronal dysfunction, suggesting that oligomeric intermediates in the aggregation process are much more toxic than fibrils (Hardy and Selkoe, 2002). In spite of substantial efforts aimed at discovering drugs against AD, no therapy has been found yet.

The BRICHOS domain is present in more than 300 proteins divided into about ten families with different functions (Hedlund et al., 2009; Sánchez-Pulido et al., 2002). The name derives from the proteins in which this domain was first discovered by sequence alignment – Bri, chondromodulin and prosurfactant protein-C (proSP-C) – and the domain consists of about 100 amino acid residues. All BRICHOS-containing proteins are suggested to be type II TM proteins, with the BRICHOS domain facing the endoplasmic reticulum (ER) lumen during biosynthesis, and they share an overall conserved architecture. Specific regions of BRICHOS-containing proteins have predicted high β-sheet propensities and, for two BRICHOS protein families, these regions are observed to form amyloid *in vivo* (Hedlund et al., 2009; Vidal et al., 1999; Vidal et al., 2000; Willander et al., 2012a). These regions are likely clients for the BRICHOS domain, e.g. the proSP-C BRICHOS domain has high affinity for the amyloidogenic TM domain of proSP-C, thereby preventing misfolding and amyloid formation (Johansson et al., 2009b; Johansson et al., 2006; Nerelius et al., 2008). The importance of the BRICHOS domain is underscored by the recent finding that mutations in the BRICHOS domain of proSP-C can give rise to interstitial lung disease with amyloid deposits composed of the proSP-C TM part (Willander et al., 2012a). Interstitial lung disease due to proSP-C BRICHOS mutations represents the first example of an amyloid disease caused by aberrant chaperone activity. Moreover, the proSP-C BRICHOS domain prevents Aβ from misfolding and aggregating into amyloid-like fibrils *in vitro* (Johansson et al., 2009b; Nerelius et al., 2009a; Nerelius et al., 2009b; Willander et al., 2012b). In the presence of BRICHOS domain, Aβ is maintained as an unstructured monomer during an extended lag phase, leading to a delay of fibril formation (Willander et al., 2012b). Notably, we recently showed that BRICHOS interferes with Aβ42 amyloid fibril formation via a mechanism that has not been described previously for any chaperone (Knight et al., 2013).

This study aimed to examine the *in vivo* effects of BRICHOS on Aβ aggregation and toxicity, and, for this purpose, *Drosophila melanogaster* was selected as the model organism (Crowther et al., 2005; Crowther et al., 2006; Greeve et al., 2004; Iijima et al., 2004). A construct containing the BRICHOS domain of proSP-C downstream of a signal peptide was incorporated into the fly genome and expressed using the Gal4/upstream activating sequence (UAS) system (Duffy, 2002). Aβ42 expression with the same system results in neuronal toxicity (Crowther et al., 2005; Greeve et al., 2004; Iijima et al., 2004). In the current study, expression was directed to neuronal cells throughout the central nervous system (CNS) by crossing the transgenic flies with the Elav^C155^-Gal4 driver fly line, which expresses Gal4 in neuronal tissue (Duffy, 2002). Flies expressing one or two transgenes coding for Aβ42, flies that express BRICHOS, and flies that co-express Aβ and BRICHOS were examined (see supplementary material Fig. S1). Longevity and locomotor activity were assayed, fly brains were analyzed by confocal microscopy after immunostaining for Aβ, and the levels of soluble and insoluble Aβ42 were determined by meso scale discovery (MSD) immunoassay. In this *Drosophila* model of AD, we show that the chaperone domain BRICHOS can efficiently reduce the neurotoxic effects of Aβ42.

**TRANSLATIONAL IMPACT**

**Clinical issue**

Protein misfolding is the underlying cause of at least 30 amyloid diseases, including prevalent disorders such as Alzheimer’s disease (AD), Parkinson’s disease and type 2 diabetes. The amyloid cascade hypothesis associated with AD posits that processing of the amyloid precursor protein produces amyloid-β peptide (Aβ), which aggregates and forms fibrils that give rise to synaptotoxicity and eventually cause dementia. Many studies indicate that pre-fibrillar intermediates, including small soluble oligomers, are more potent than monomers or fibrils in causing neuronal dysfunction, suggesting that oligomeric intermediates in the aggregation process are much more toxic than fibrils. Despite substantial efforts in AD drug discovery, effective therapies are not currently available. This paper focuses on the recently discovered chaperone domain BRICHOS, which is associated with amyloid disease. Recent *in vitro* data showed that this domain efficiently delays Aβ42 oligomerization and fibril formation. The present study aimed to examine the *in vivo* effects of BRICHOS on Aβ aggregation and in particular its toxicity, using *Drosophila melanogaster* as the model organism.

**Results**

The authors generated transgenic flies that express the Aβ42 peptide and the BRICHOS domain in the central nervous system. Co-expression of Aβ42 and BRICHOS resulted in delayed Aβ42 aggregation and dramatic improvements in both lifespan and locomotor function compared with flies expressing Aβ42 alone. Moreover, the authors analyzed the Aβ42 protein levels in the fly brains and showed that BRICHOS increases the ratio of soluble to aggregated Aβ42. Finally, using immunostaining of whole fly brain mounts and confocal microscopy, the authors demonstrated that BRICHOS co-expression delays but does not prevent aggregation of Aβ42.

**Implications and future directions**

These results indicate that the chaperone domain BRICHOS reduces Aβ42 toxicity by a novel mechanism – it blocks a major source of toxic Aβ oligomers by diverting fibril formation to a slower pathway, rather than reducing the overall aggregation. Thus, neurotoxicity is ameliorated although Aβ42 aggregation continues to occur. These findings suggest that BRICHOS-based approaches hold promise for the prevention and treatment of AD. Further studies in different *Drosophila* and mouse models of AD are needed to fully characterize the effects of BRICHOS administration on disease progression and toxicity.

## RESULTS

### Generation and characterization of fly lines

The fly lines containing single and double copies of a signal-peptide–*Aβ42* transgene were generated as described (Crowther et al., 2005). We generated a fly line that contains a signal peptide followed by the linker region and BRICHOS domain of proSP-C (see supplementary material Fig. S1), referred to as BRICHOS hereafter, in order to enable the expression of both Aβ42 and BRICHOS in the secretory pathway. The different fly lines were crossed with each other and/or with driver fly lines expressing Gal4 in the CNS, in order to obtain flies that express single or double copies of the *Aβ42* transgene (Aβ42×1 and Aβ42×2, respectively), BRICHOS alone, or single or double copies of the *Aβ42* transgene together with BRICHOS (Aβ42×1 + BRICHOS and Aβ42×2 + BRICHOS). The expression of Aβ42 and BRICHOS in different combinations is all driven by the Gal4 expression from the Elav^C155^-Gal4 driver strain. The control flies expressed Gal4 only, under the same promoter. In order to make sure that the individual expression levels were not altered by simultaneous expression of several genes, the Aβ42 expression in the different fly lines was analyzed by real-time quantitative PCR. The results showed that simultaneous expression of Aβ42 and BRICHOS, under the *Gal4* driver, does not affect *Aβ42* mRNA levels (see supplementary material Fig. S2).

We further investigated whether the insertion of the different transgenes, i.e. UAS-Aβ42×1, UAS-Aβ42×2 and UAS-BRICHOS, as such affected the longevity or locomotor activity. In the longevity experiments, 100 W1118 and 100 each of the transgenic flies were studied, and, in the locomotor assay, 50 flies of each line were studied (see supplementary material Fig. S3). This shows that the insertions of the transgenes as such do not give rise to any significant effects on the phenotypic traits studied (see supplementary material Fig. S3 and Table S1).

### BRICHOS significantly improves the longevity of Aβ42-expressing flies

**Fig. 1.**
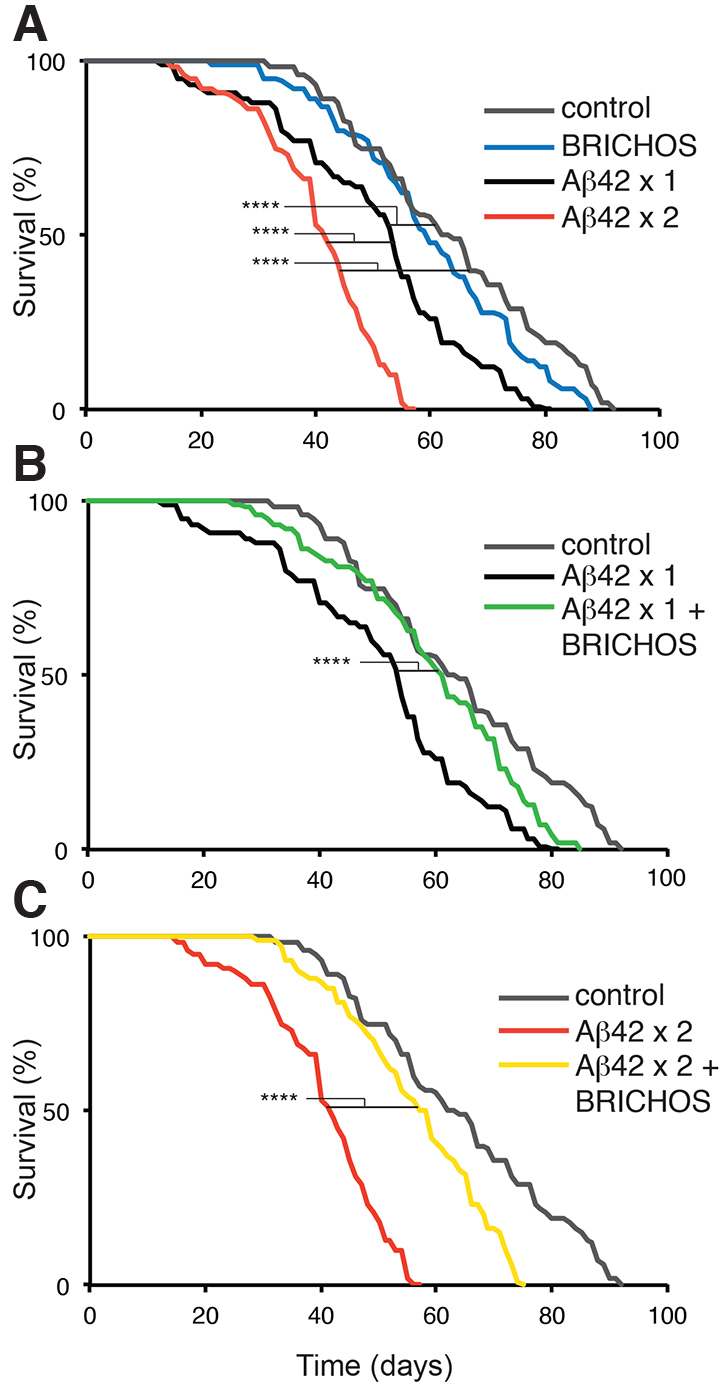
**BRICHOS suppresses the toxic effects of Aβ42 on *Drosophila* lifespan.** The fraction of 100 living flies over time is plotted for (A) control flies, and flies expressing BRICHOS, Aβ42×1 and Aβ42×2; (B) control flies, and flies expressing Aβ42×1 and Aβ42×1 + BRICHOS; and (C) control flies (Gal4-*elav*^c155^), and flies expressing Aβ42×2 and Aβ42×2 + BRICHOS. Survival plots were calculated using the Kaplan-Meier method and differences between groups were tested using the log-rank test, ****P<0.0001. See supplementary material Table S2 for statistical analyses of differences between all groups.

Flies expressing BRICHOS, Aβ42×1, Aβ42×2, Aβ42×1 + BRICHOS or Aβ42×2 + BRICHOS, and control flies (expressing Gal4) were analyzed for longevity at a temperature of 25°C. The lifespan of 100 flies of each genotype was measured. The results show that flies with neuronal expression of Aβ42×1 (median lifetime 54 days) have a statistically significant decrease in lifespan when compared with control flies (median lifetime 63 days) (*P*<0.0001). Flies with double copies of the *Aβ42* gene have a median lifetime of 42 days (*P*<0.0001 vs control flies) (Fig. 1A), indicating that the Aβ42-induced effects on longevity are dose-dependent. The longevity of Aβ42-expressing flies, for both expression levels, was significantly increased by co-expression of the BRICHOS domain, resulting in a median lifespan of 62 and 58 days for Aβ42×1 + BRICHOS and Aβ42×2 + BRICHOS flies, respectively (*P*<0.0001) (Fig. 1B,C; see supplementary material Table S2). Flies expressing BRICHOS alone had a median lifespan of 60 days, similar to the control flies (Fig 1A; see supplementary material Table S2). This shows that, independent of Aβ42 expression levels, BRICHOS has robust effects on Aβ42 toxicity in the fly model. In order to further study the BRICHOS effects on Aβ42 toxicity (measured as locomotor activity) and aggregation (studied by confocal microscopy and MSD immunoassay), we focused on the time period up to 25 days, during which the longevity assay showed that BRICHOS had detectable effects on the survival of Aβ42×1 and Aβ42×2 flies (Fig. 1).

### BRICHOS improves the locomotor activity of Aβ42-expressing flies

**Fig. 2.**
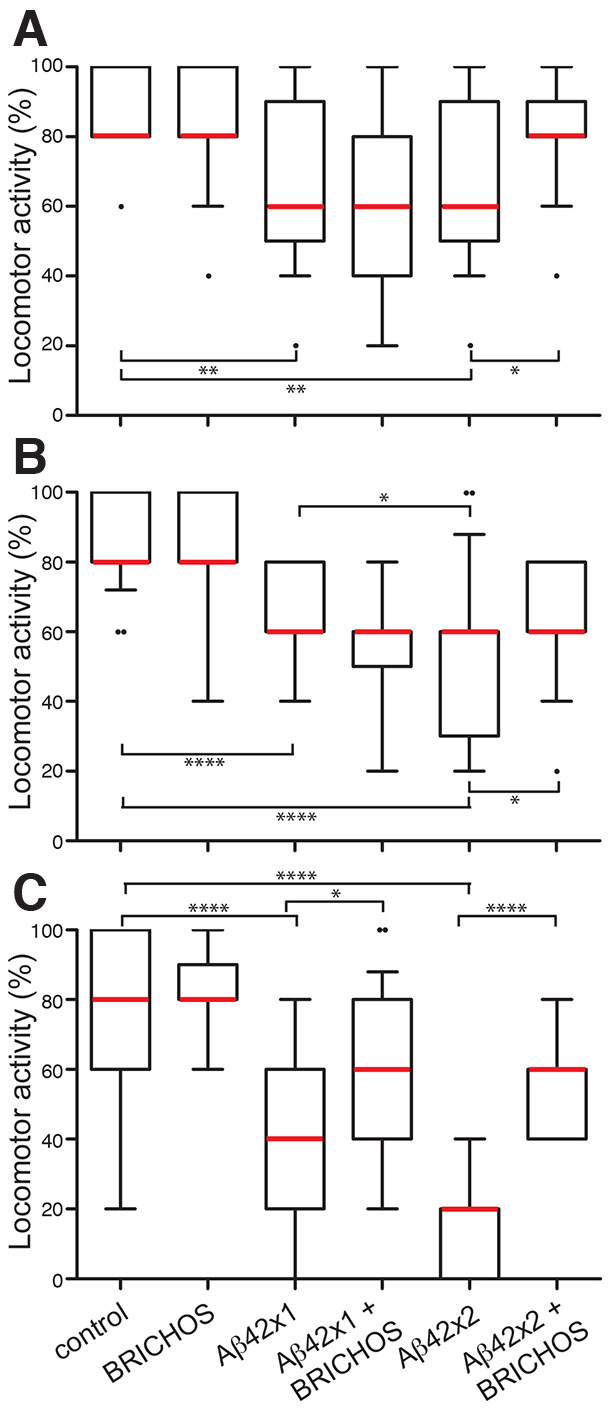
**BRICHOS reduces the toxic effects of Aβ42 on locomotor activity.** Aβ42×1, Aβ42×2, Aβ42×1 + BRICHOS, Aβ42×2 + BRICHOS and control (Gal4-*elav*^c155^) flies were analyzed in a climbing assay. Flies were analyzed at (A) day 10, (B) day 15 and (C) day 25. The number of flies passing a line (8 cm above ground) within 10 seconds was counted and expressed as a percentage for each group of five flies. Twenty-five flies of each genotype divided into five tubes were analyzed five times, and statistical analyses were made using the Mann-Whitney test. The boxes extend from the 25th to 75th percentiles, the whiskers are drawn from the 10th percentile to the 90th percentile and the black dots represent outliers. The red line represents the median value. ****P<0.0001, **P<0.01, *P<0.05. See supplementary material Table S2 for statistical analyses of differences between all groups.

Locomotor activity was assessed for 10-, 15- and 25-day-old flies in a climbing assay (Crowther et al., 2006; Nerelius et al., 2009b). For each type of fly, the number of flies that reach 8 cm high during a 10-second observation period was counted five times for 25 flies in groups of five. Expression of Aβ42 alone is known to result in reduced climbing activity (Crowther et al., 2005; Nerelius et al., 2009b). Here, we observed Aβ42 dose- and time-dependent reductions in locomotor activity: there was a significant reduction from day 10 and activity was severely impaired at day 25 in particular for Aβ42×2-expressing flies (Fig. 2; see supplementary material Table S2). At all time points, Aβ42×2 + BRICHOS flies show a locomotor activity that is significantly higher than that of Aβ42×2 flies, whereas expression of BRICHOS only had no significant effect on climbing ability compared with control flies (Fig. 2; see supplementary material Table S2). The Aβ42×1-expressing flies showed decreased locomotor activity already from day 10, and improvement by BRICHOS was observed at day 25, when the locomotor deficiency was more severe (Fig. 2).

### BRICHOS delays Aβ42 deposition in the fly brain

**Fig. 3.**
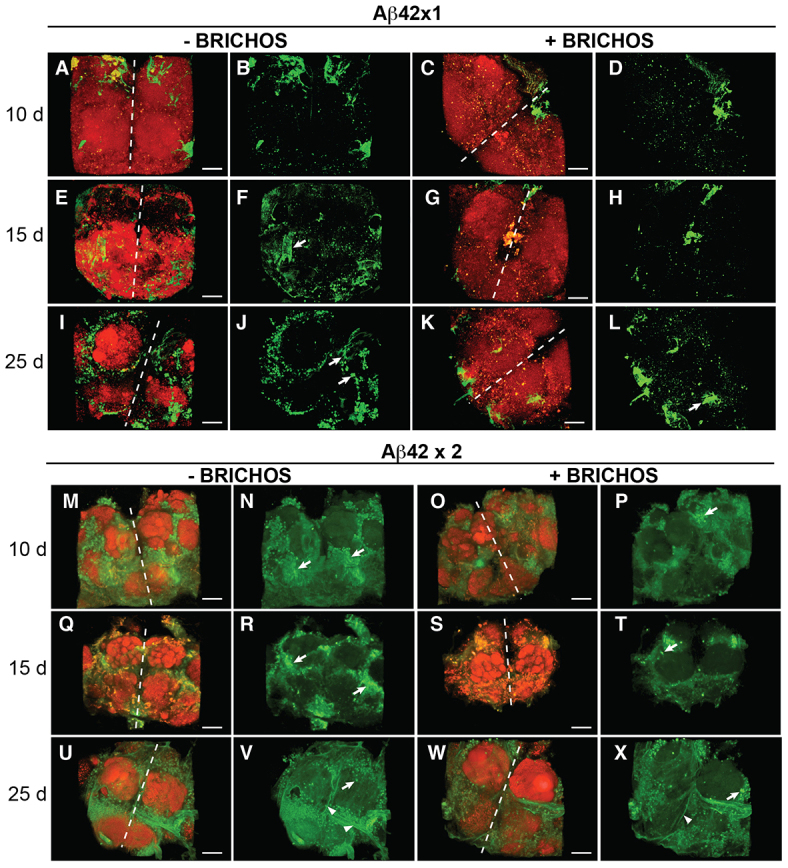
**BRICHOS delays Aβ42 deposition in the brain.** Confocal images of fly brains immunostained with an anti-Aβ42 antibody (green) and for presynaptic zones (bruchpilot antibody; red), obtained at age 10, 15 and 25 days: Aβ42×1 (A,B,E,F,I,J), Aβ42×1 + BRICHOS (C,D,G,H,K,L), Aβ42×2 (M,N,Q,R,U,V), Aβ42×2 + BRICHOS (O,P,S,T,W,X). For each specimen, two figures are shown: the one to the right showing Aβ42 staining only and the one to the left showing Aβ42 staining merged with presynaptic-zone staining. Arrows mark examples of dense granular deposits and arrowheads mark examples of fiber-like structures. The results are representative for three independent experiments. Scale bars: 40 μm. The white dotted line marks the border between the two brain hemispheres. The localization in the fly brain of the area shown here is given in supplementary material Fig. S4A.

Brain tissue from control, Aβ42×1, Aβ42×2, Aβ42×1 + BRICHOS and Aβ42×2 + BRICHOS flies were stained with an Aβ42-specific antibody and examined with confocal microscopy. The flies were examined at the same time points as in the locomotor studies. Already at day 10 the Aβ42×2 flies showed abundant dense, punctuate depositions of Aβ and, at day 25, the deposits were larger, continuous and partly fiber-like (Fig. 3N,R,V). Aβ42×1 flies showed Aβ depositions at all time points, but these deposits were less abundant than for the double-expressing flies and did not cover major parts of the brain until day 25 (Fig. 3B,F,J). Co-expression of BRICHOS resulted in reduced Aβ42 staining at all time points for the single-expressing flies (Fig. 3D,H,L) and, for days 10 and 15, for the double-expressing flies (Fig. 3P,T). Aβ42×2 + BRICHOS flies showed a slightly reduced Aβ42 deposition compared with Aβ42×2 flies at day 25, but also, in the presence of BRICHOS, the Aβ42 deposits at this point were dense and covered a large part of the brain (Fig. 3X).

### BRICHOS increases soluble Aβ42 and decreases aggregated Aβ42

**Fig. 4.**
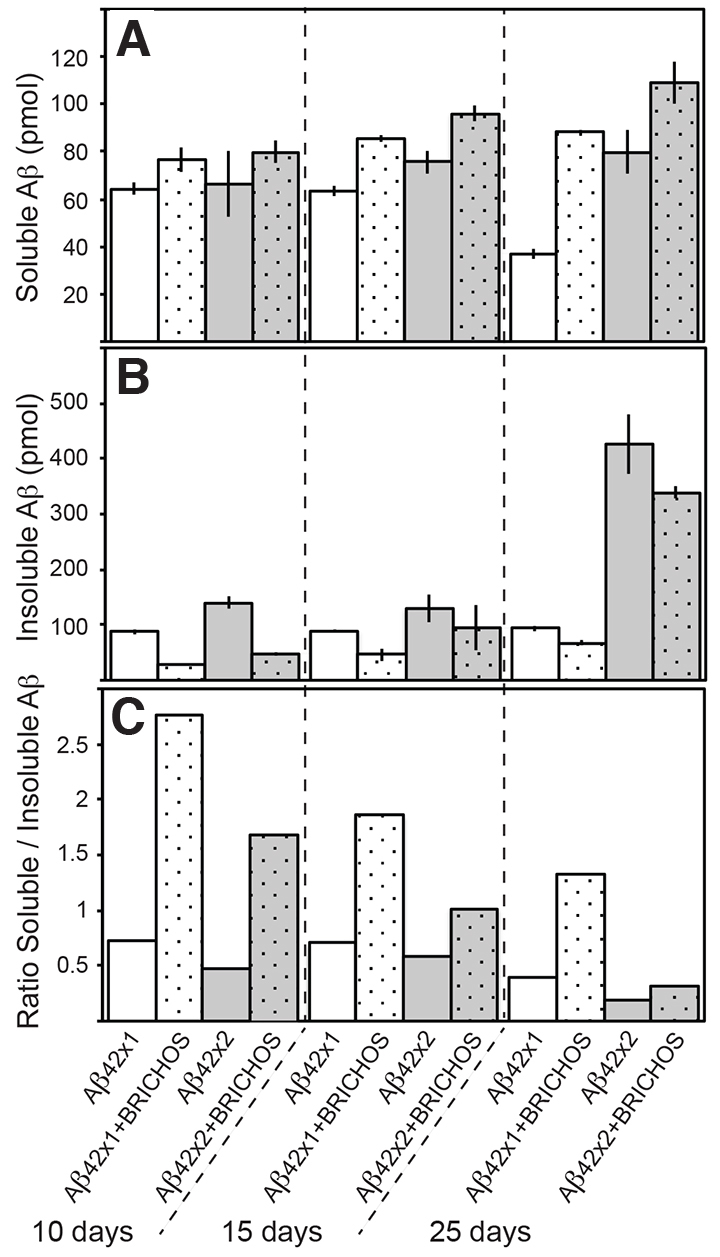
**BRICHOS delays aggregation of Aβ42 and increases soluble Aβ42 in the fly brain.** Quantification of soluble and insoluble Aβ42 protein, from five fly heads of each of the different groups studied, was determined using MSD immunoassay. The levels of soluble Aβ42 in HEPES buffer pH 7.3 (A) and the levels of insoluble Aβ42 (dissolved using 5 M guanidinium-HCl in HEPES buffer pH 7.3) (B) (referred to as insoluble in the text), as well as the ratio (C) between soluble and insoluble Aβ, were plotted for day 10, 15 and 25 for the different groups. Error bars in A and B correspond to the standard deviation of the sample. The results are representative for three independent experiments.

Confocal immunofluorescence imaging of the fly brains (Fig. 3) showed Aβ42 deposits both with and without co-expression of the BRICHOS domain, with a small reduction seen in the flies that co-expressed BRICHOS. In order to analyze whether the amounts of soluble or insoluble Aβ were altered by BRICHOS co-expression, fly brain homogenates in HEPES buffer pH 7.3 without or with 5 M guanidinium-HCl, respectively, were analyzed by MSD immunoassay for Aβ42. As a result of BRICHOS expression, the levels of soluble Aβ42 were increased, whereas the levels of insoluble Aβ42 were decreased and, as a consequence, the ratio of soluble:insoluble Aβ42 increased, at all time points (Fig. 4). The ratio of soluble:insoluble Aβ42 decreased over time for all types of flies (Fig. 4C). At day 25 the Aβ42×2- and Aβ42×2 + BRICHOS-expressing flies showed a distinct increase in insoluble Aβ levels, compared with the same flies at days 10 and 15, and compared with Aβ42×1 flies at all time points (Fig. 4A,B). It is possible that this reflects the appearance of larger, fiber-like deposits seen by confocal microscopy in brains of Aβ42×2 and Aβ42×2 + BRICHOS flies at day 25 (Fig. 3V,X).

### BRICHOS and Aβ42 co-deposits in fly brain as well as *in vitro*

**Fig. 5.**
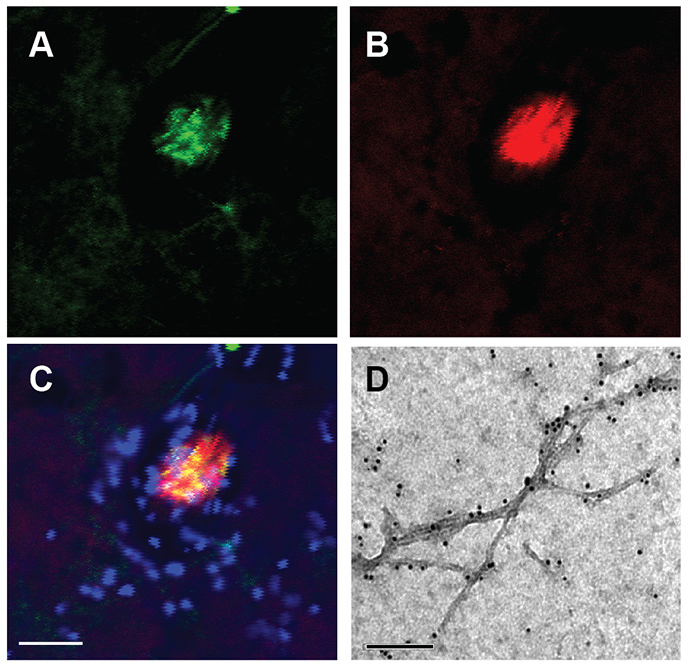
**BRICHOS colocalizes with Aβ42 in the fly brain and binds to Aβ42 fibrils formed *in vitro*.** Immunostaining for (A) Aβ42 (6E10 antibody; green), (B) BRICHOS (anti-BRICHOS antiserum; red) and (C) both Aβ42 and BRICHOS in the brain of an Aβ42×1 + BRICHOS fly, using confocal microscopy at 63× magnification, with DAPI (blue) as counterstaining for nuclei. (D) 3 μM of Aβ42 was co-incubated with 0.7 molar equivalents of recombinant BRICHOS at 37°C overnight and an aliquot was analyzed by immuno-gold labeling for BRICHOS and electron microscopy. Scale bars: (C) 20 μm; (D) 200 nm.

Immunofluorescence staining showed that Aβ42 and BRICHOS colocalize in the fly brain (Fig. 5A–C). Moreover, fibrils of Aβ42 that formed *in vitro* in the presence of recombinant human BRICHOS domain were decorated by BRICHOS, as shown by immuno-gold labeling and electron microscopy (Fig. 5D).

## DISCUSSION

Analyses of lifespan and locomotor activity show that BRICHOS expression in the CNS is well tolerated by the flies and does not give rise to any detectable toxic effects (Figs 1, 2). In contrast, expression of Aβ42×1 or Aβ42×2 gives rise to significant shortening of lifespan and reduced locomotor activity compared with control flies expressing the Gal4 protein only, in a dose-dependent manner (Figs 1, 2), in line with previous observations (Crowther et al., 2005; Speretta et al., 2012). BRICHOS delays the accumulation of Aβ42 according to immunofluorescence analyses and MSD immunoassay (Figs 3, 4), and BRICHOS almost completely prevents the toxic effects of Aβ42 on longevity and locomotor activity (Figs 1, 2). This shows that presence of the BRICHOS domain delays Aβ42 aggregation *in vivo* and, importantly, that BRICHOS decreases Aβ42 toxicity even when significant aggregation of Aβ42 is observed (see below). Recently, it was shown, using the same Aβ42 transgenic fly model as we used in this study, that Aβ deposition and toxicity could be reduced by co-expression of an engineered high-affinity Aβ-binding affibody (Z_Aβ3_). Aβ was apparently sequestered from the fly brain by Z_Aβ3_ co-expression, via an unknown mechanism (Luheshi et al., 2010). Transgenic *Drosophila* has been used to show that the molecular chaperone Hsp70 can suppress toxic effects in models of neurodegenerative diseases (polyglutamine-repeat disease and Parkinson’s disease) (Auluck et al., 2002; Warrick et al., 1999). However, to the best of our knowledge this study is the first demonstration of prevention of toxic effects of Aβ42 expression in flies by a chaperone-like domain.

The Aβ42 aggregation mechanism was recently found to include a feedback reaction in the form of secondary nucleation, whereby Aβ monomers associate to the surface of already existing fibrils and form new oligomers, some of which are toxic (Cohen et al., 2013). Here, we show that BRICHOS delays the formation of insoluble Aβ42 aggregates and limits the formation of toxic forms of Aβ42 (Figs 1–4). Moreover, colocalization studies suggest that BRICHOS binds to Aβ42 aggregates and fibrils (Fig. 5).

BRICHOS efficiently delays both toxicity (Figs 1, 2) and aggregation of Aβ42 (Figs 3, 4) in the Aβ42×1-expressing flies. In contrast, the Aβ42×2 + BRICHOS flies showed abundant aggregation of Aβ42 at day 25, similar to that of Aβ42×2 flies (Fig. 3V,X; Fig. 4C). Importantly, however, the lifespan of Aβ42×2 + BRICHOS flies was not significantly different from that of flies expressing BRICHOS only (Fig. 1; supplementary material Table S2), and the locomotor activity of Aβ42×2 + BRICHOS flies at day 25 was dramatically improved compared with Aβ42×2 flies (Fig. 2C). This discrepancy between Aβ aggregation and toxicity at late time points suggests that BRICHOS interferes with the generation of toxic species rather than the formation of mature fibrils. This suggests that BRICHOS can be an effective agent against Aβ toxicity even if Aβ aggregation and fibril formation is only delayed (Willander et al., 2012b; Knight et al., 2013).

Recent results from transgenic mice overexpressing Aβ42 as a fusion protein with the Bri2 protein support the possibility that BRICHOS prevents Aβ toxicity despite only delaying, rather than preventing, amyloid fibril formation. In this study (Kim et al., 2013), the normal C-terminal peptide of Bri2, Bri23, was substituted for Aβ42, which was released by proteolysis to generate free Aβ42. Surprisingly, these mice were not at all cognitively affected, even though they had high Aβ42 expression and eventually developed amyloid plaques. The authors suggested that high Aβ42 levels and aggregates are not sufficient to induce memory dysfunction, and that derivatives of APP processing (which were obviously not generated from the Bri2-Aβ construct used) are contributing to the toxicity seen in APP transgenic mouse models (Kim et al., 2013). However, the Bri2 BRICHOS domain has previously been shown to be released from its proprotein by proteolysis (Martin et al., 2008), and an alternative explanation to the lack of toxic effects seen in the Bri2-Aβ42-expressing mouse model (Kim et al., 2013) could be that liberated BRICHOS domain delays Aβ aggregation and prevents toxicity, in a similar manner as now observed in our fly model. This possibility is further supported by the fact that the Bri2-Aβ-expressing mice formed much fewer Aβ oligomers than APP-expressing mice (Kim et al., 2013), a finding that is difficult to explain by the absence of APP-processing products. To explore whether BRICHOS can be harnessed for future treatment of AD, it will be necessary to further analyze the effects of BRICHOS co-expression and administration in different *Drosophila* and mouse models of Aβ42 aggregation and toxicity.

## MATERIALS AND METHODS

### Transgenic Drosophila melanogaster

The cDNA sequence of human proSP-C BRICHOS (proSP-C_59–197_, NP_001165881), including an upstream sequence for the human surfactant protein B (SP-B_1–23_, NP_000533) signal peptide was inserted into the pUASTattB vector and site-specific transgenesis to the 86Fb locus on chromosome 3 was achieved using the φC31 system (BestGene Inc., CA). Two fly lines transgenic for human Aβ42 plus a signal peptide from the *Drosophila* necrotic gene (NP_524851), one with the transgene inserted in chromosome 2 and one with the transgene inserted in chromosomes 2 and 3, were used in order to obtain single and double expression of Aβ42, respectively (Aβ42×1 and Aβ42×2, respectively) (Crowther et al., 2005). These flies were crossed with BRICHOS-expressing flies to generate Aβ42×1 + BRICHOS and Aβ42×2 + BRICHOS flies. Aβ42 and BRICHOS protein was expressed by crossing transgenic flies with the Gal4-*elav*^c155^ pan-neuronal driver strain (Duffy, 2002). W1118 wild-type flies were crossed with the Gal4-*elav*^c155^ driver strain, which gave rise to flies with pan-neuronal expression of Gal4, used as control flies.

### Longevity assay

Flies expressing BRICHOS, Aβ42×1, Aβ42×2, Aβ42×1 + BRICHOS or Aβ42×2 + BRICHOS, or control flies (W1118, expressing Gal4 only), under the influence of the pan-neuronal driver Gal4-*elav*^c155^ were analyzed at a temperature of 25°C and 55% humidity. Offspring female flies were collected and divided into ten tubes with each tube containing ten flies. The food in these tubes was changed and the number of living flies was counted every 2–3 days, until there were no living flies left. The percentage of living flies for each type of strain was plotted against age of flies. Survival plots were calculated using the Kaplan-Meier method and differences between groups was tested using the log-rank test (GraphPad Prism). In order to rule out effects of transgene insertion as such, longevity of UAS-Aβ42×1, UAS-Aβ42×2 and UAS-BRICHOS (all lacking Gal4 driver) crossed with W1118 was analyzed at 26°C and 70% humidity.

### Locomotor activity

Five flies were placed in an empty vial with a line marked at 8 cm from the bottom. The tube was dropped from 10 cm height onto a hard surface and the number of flies that passed the 8 cm line in 10 seconds was determined, using a Canon 5D camera. For the expressing flies, 25 flies per group were used and separated into five tubes containing five flies. Each tube was analyzed five times, where the number of flies that passed the line was counted, and the median values of 25 (5×5) locomotor assays of five flies per assay were calculated for each group. Statistical analyses were made by using the Mann-Whitney test (GraphPad Prism). Locomotor activity was also measured for non-expressing transgenes of UAS-Aβ42×1, UAS-Aβ42×2 and UAS-BRICHOS crossed with W1118. In this experiment, flies were reared at 26°C and 50 flies of each strain were analyzed.

### MSD Aβ42 analysis

Five fly heads of each group were put on ice in 50 μl extraction buffer containing 50 mM HEPES, pH 7.3, 5 mM EDTA and Complete EDTA-free Protease Inhibitor (Roche). The heads were homogenized with a handheld homogenizer (VWR) for 1 minute, followed by vortexing. The samples were then sonicated for 4 minutes followed by a centrifugation at 13,000 rpm for 5 minutes. The supernatant was diluted ten times with 25 mM HEPES buffer, pH 7.3, 1 mM EDTA, 0.1% Blocker A (Meso Scale Discovery, MD), and referred to as the soluble fraction. The insoluble material from the pellet was dissolved in 50 μl of the same buffer containing 5 M guanidinium-HCl (GuHCl), and homogenized for 1 minute followed by vortexing. The GuHCl-dissolved samples were then sonicated for 4 minutes and centrifuged at 13,000 rpm for 5 minutes, and the supernatant was transferred to a new tube and diluted ten times, and referred to as the insoluble fraction.

Soluble and insoluble Aβ42 levels were then measured using MSD 96-Well MULTI-ARRAY Human (6E10 antibody) Aβ42 Ultra-Sensitive Kit (K151FUE-2, Meso Scale Discovery, MD) as described (Caesar et al., 2012). Aβ42 standard peptide solution, provided in the MSD kit, was dissolved as described in MSD protocol with concentrations between 4.1 and 3000 pg/ml. The samples plate was first blocked with 25 μl 10% Blocker A solution for 30 minutes at 22°C with slow shaking, and then washed three times with Tris Wash Buffer (Meso Scale Discovery, MD). Twenty-five μl of samples or standard peptide were added to each well and incubated with shaking for 1 hour, and washed again. Twenty-five μl SULFO-TAG detection antibody (Meso Scale Discovery, MD) was added to each well and incubated with shaking for 1 hour, followed by washing. Then, 150 μl 2× Read Buffer (Meso Scale Discovery, MD) was added to each well and the plate was analyzed using a SECTOR Imager 2400. The Aβ42 levels of each group were normalized using the total protein concentrations of the head homogenates, as measured by the Bio-Rad protein assay kit.

### Immunofluorescence staining

For whole brain mounts, fly heads were collected and immersed in 70% ethanol for 30 seconds, transferred to PBS and brains were then dissected under a light microscope. Whole brains were then fixed for 15 minutes in 4% paraformaldehyde on ice, washed in PBS-T (0.2% Triton X-100 in PBS) for 3×10 minutes and blocked in 5% BSA in PBS-T for 1 hour at room temperature (RT). Primary antibodies used were mouse anti-Aβ1–16 (6E10, Nordic BioSite), rabbit anti-proSP-C C-terminal antisera (Johansson et al., 2009a), rabbit anti-β-amyloid-42 (Invitrogen) and mouse anti-bruchpilot (nc82, DSHB). Primary antibodies were diluted 1:1000 in blocking buffer and incubated with brain samples for 24 hours, followed by wash with PBS-T for 3×10 minutes. Secondary antibodies (Molecular Probes) of goat anti-mouse Alexa Fluor 488, goat anti-mouse Alexa Fluor 555 or goat anti-rabbit Alexa Fluor 633 were diluted 1:1000 in blocking buffer and incubated with the specimens for 1 hour at RT. Samples were then washed in PBS-T for 3×10 minutes and mounted with either DAPI mounting media (Vectashield, Vector Laboratories Inc.) or in 1:1 PBS and 85% glycerol. Images were collected with a confocal microscope (Zeiss, LSM 520) with 10×, 40× or 63× magnification objectives using single-plane or Z-stack settings. Images were visualized, processed and 3D-reconstructed using ImageJ software (National Institutes of Health, MD).

### Real-time quantitative PCR

Five fly heads of 5-day-old flies of each genotype were collected and mRNA was extracted using Oligotex mRNA kit (Qiagen). cDNA synthesis from 100 ng of mRNA was performed using a High Capacity RNA to cDNA kit (Applied Biosystems), according to the manufacturer’s protocol. Real-time quantitative PCR was performed using SYBR Green PCR Master Mix (Applied Biosystems), 10 ng of cDNA template and 100 nM of primer pairs, and analyzed with 7500 Fast Real-Time PCR System (Applied Biosystems). Each sample was run in triplicates. Primers for Aβ42 were 5′-CAGC-GGCTACGAAGTGCATC-3′ (forward) and 5′-CGCCCACCATCAAGCC-AATA-3′ (reverse); primers for the housekeeping gene (RP 49) were 5′-ATGACCATCCGCCCAGCATCAG-3′ (forward) and 5′-ATCTCGCC-GAGTAAACG-3′ (reverse).

### Transmission electron microscopy

3 μM of recombinant Aβ42 (Walsh et al., 2009) with 0.7 equivalents of recombinant BRICHOS (Willander et al., 2012a) were incubated at 37°C overnight. Aliquots of 2 μl were loaded on Nickel-coated grids, and excess of sample was removed. The grids were placed on a drop of 1% BSA in TBS, incubated for 30 minutes at RT, and then washed for 10 minutes on each of three drops of TBS. The grids were then placed on drops of rabbit anti-C-terminal proSP-C antibody diluted 1:200 in TBS, and incubated overnight at +4°C. After washing 10 minutes on each of five drops, the grids were placed on a drop of goat anti-rabbit IgG coupled to 10-nm gold particles diluted 1:40 in TBS, and incubated for 2 hours at RT. The grids were then washed five times as before, followed by negative staining with 2% uranyl acetate in 50% ethanol. The immunolabeled fibrils were examined using a Hitachi H7100 TEM operated at 75 kV.

## Supplementary Material

Supplementary Material
