# Supplementary material for: The chaperone domain BRICHOS prevents CNS toxicity of amyloid-β peptide in Drosophila melanogaster
Source: Dis Model Mech. 2014 Mar 28;7(6):659–65. doi: 10.1242/dmm.014787 (PMC4036473; doi:10.1242/dmm.014787)
Supplement: Supplementary Material [file supp_7_6_659__index.html]

The chaperone domain BRICHOS prevents CNS toxicity of amyloid-β peptide in Drosophila melanogaster — Supplementary Material 

# The chaperone domain BRICHOS prevents CNS toxicity of amyloid-β peptide in *Drosophila melanogaster*

## DMM014787 Supplementary Material

**Files in this Data Supplement:**

- **Supplementary Material**
