# Supplementary material for: The chaperone domain BRICHOS prevents CNS toxicity of amyloid-β peptide in Drosophila melanogaster
Source: Dis Model Mech. 2014 Mar 28;7(6):659–65. doi: 10.1242/dmm.014787 (PMC4036473; doi:10.1242/dmm.014787)
Supplement: Supplementary Material [file supp_7.6.659_DMM014787.pdf]

Supplemental information

The chaperone domain BRICHOS prevents amyloid β-peptide CNS toxicity in Drosophila melanogaster

Erik Hermansson[1], Sebastian Schultz[2], Damian Crowther[3], Sara Linse[4], Bengt Winblad[1], Gunilla Westermark[5], Jan Johansson[1,6,7*], Jenny Presto[1*]

[1]KI Alzheimer Disease Research Centre, NVS Department, Karolinska Institutet, Novum, 5th floor, 141 86 Stockholm, Sweden, [2]Department of Biochemistry, Institute for Cancer Research, Oslo University Hospital–The Norwegian Radium Hospital, 0379 Oslo, Norway, [3]Department of Genetics, University of Cambridge, Downing Street, Cambridge CB2 3EH, UK, [4]Department of Biochemistry and Structural Biology, Lund University, P. O. Box 124, SE221 00 Lund, Sweden, [5]Department of Medical Cell Biology, Uppsala University, 751 23 Uppsala, Sweden [6]Department of Anatomy, Physiology and Biochemistry, Swedish University of Agricultural Sciences, The Biomedical Centre, 751 23 Uppsala, Sweden. [7]Institute of Mathematics and Natural Sciences, Tallinn University, Narva mnt 25, 101 20 Tallinn, Estonia.

*Corresponding authors: janne.johansson@ki.se, jenny.presto@ki.se

Table S1: Lack of effects on longevity and locomotor activity of Aβ42 or BRICHOS transgenes. Survey of p values for between-groups comparisons of longevity (in italics in the upper right side) and locomotor activity (underlined in the lower left side).

	**W1118**	**UAS-Aβ42x1**	**UAS-Aβ42x2**	**UAS-BRICHOS**
**W1118**		*0.6032*	*0.7709*	*0.2461*
**UAS-Aβ42x1**	0.7527		*0.3286*	*0.0683*
**UAS-Aβ42x2**	0.3071	0.1649		*0.3913*
**UAS-BRICHOS**	0.6271	0.4092	0.5552	

Log-rank test was used to calculate p values between groups for longevity measurements, and Mann Whitney test was used to calculate the p values between groups for locomotor activity measurements at day 25.

Table S2: Effects on longevity and locomotor activity of Aβ42 and/or BRICHOS expression. Survey of p values for between-groups comparisons of longevity (in italics in the upper right side) and locomotor activity (underlined in the lower left side).

	**Control (Gal4-*elav*^{c155})**	**BRICHOS**	**Aβ42x1**	**Aβ42x2**	**Aβ42x1 + BRICHOS**	**Aβ42x2 BRICHOS**
**Control (Gal4-*elav*^{c155})**		*0.1831*	*< 0.0001*	*< 0.0001*	*0.1327*	*0.0019*
**BRICHOS**	0.375		*< 0.0001*	*< 0.0001*	*0.8403*	*0.0602*
**Aβ42x1**	< 0.0001	< 0.0001		*< 0.0001*	*< 0.0001*	*0.0133*
**Aβ42x2**	< 0.0001	< 0.0001	< 0.0001		*< 0.0001*	*< 0.0001*
**Aβ42x1 + BRICHOS**	0.0188	0.0002	0.0143	< 0.0001		0.0615
**Aβ42x2 + BRICHOS**	0.0027	< 0.0001	0.0074	< 0.0001	0.8703	

Log-rank test was used to calculate p values between groups for longevity measurements, and Mann Whitney test was used to calculate the p values between groups for locomotor activity measurements at day 25.

Supplementary figure legends

Supplementary figure 1. **Gene constructs inserted into the transgenic *Drosophila melanogaster* flies.** (A) The coding sequence for human Aβ42, corresponding to position 672-713 in human APP, downstream of the signal peptide of *Drosophila* necrotic gene was inserted in the 2nd and/or the 3rd chromosome. The sequence coding for the linker and BRICHOS domain of proSP-C downstream of the signal peptide of human proSP-B gene was inserted at the 3rd chromosome. (B) Crossings were made so that the flies containing the genotypes Aβ42x1, Aβ42x2, BRICHOS, Aβ42x1 + BRICHOS, and Aβ42x2 + BRICHOS were obtained, which were then crossed with the $Elav^{C155}$-Gal4 driver fly line for pan-neuronal expression.

Supplementary figure 2. **BRICHOS expression does not affect Aβ42 gene expression levels.** $Elav^{C155}$-Gal4 induced Aβ42 mRNA expression levels in BRICHOS co-expressing flies was compared to levels in Aβ42 only expressing flies using quantitative RT-PCR. (A) Relative mRNA levels in Aβ42x1 and Aβ42x1 + BRICHOS flies, and (B) relative mRNA levels of Aβ42 in Aβ42x2 and Aβ42x2 + BRICHOS flies.

Supplementary figure 3. **Lack of effects on longevity and locomotor activity of Aβ42 or BRICHOS transgenes.** (A) The fraction of 100 living flies over time is plotted for W1118 wildtype flies, UAS-Aβ42x1, UAS-Aβ42x2 and UAS-BRICHOS. Survival plots were calculated using the Kaplan-Meier method. (B) W1118 wildtype flies, UAS-Aβ42x1, UAS-Aβ42x2 and UAS-BRICHOS flies were analyzed in a climbing assay. Flies were analyzed at day 25 and the number or flies passing a line (8 cm above ground) within 10 sec were counted and expressed as percentage for each group of five flies. 50 flies of each genotype divided in 10 tubes were analyzed five times, and statistical analyses were made using the Mann Whitney test. The boxes extend from 25th to 75th percentiles and the whiskers are drawn from the 10th percentile to the 90th percentile.

Supplementary figure 4. **Negative controls for the confocal analyses.** (A) A dissected brain from a Gal4-*elav*^{c155} control fly was immunostained with anti-Aβ42 antibody (green) and for

presynaptic zones (bruchpilot, red) and analyzed with confocal microscopy. The marked area in (A) is enlarged in images (B) to (E). (B) Only Aβ42 staining, (C) staining for Aβ42 and presynaptic zones. (D) Shows the staining using only the secondary antibody for anti-Aβ42 (goat anti-rabbit Alexa Fluor 488) and (E) shows the same staining together with staining for presynaptic zones. Scale bar in (A) represents 200 mm.

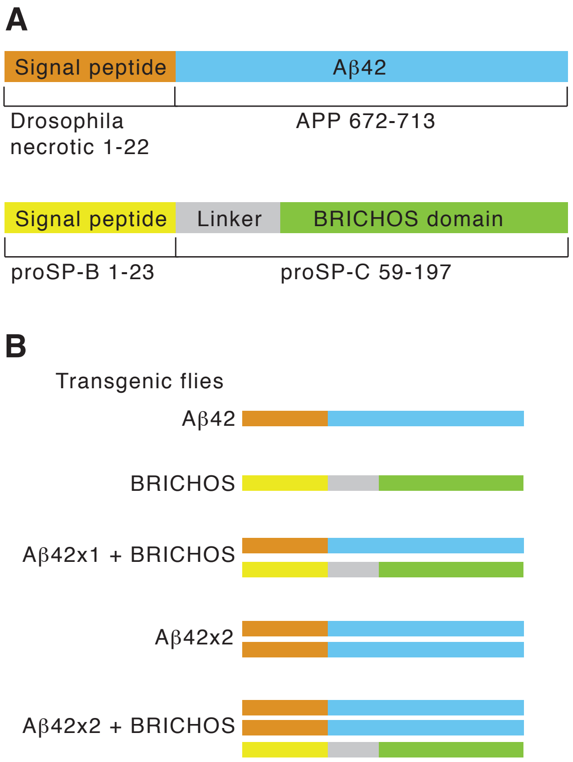
A
Signal peptide
Aβ42
Drosophila
necrotic 1-22
APP 672-713
Signal peptide
Linker
BRICHOS domain
proSP-B 1-23
proSP-C 59-197
B
Transgenic flies
Aβ42
BRICHOS
Aβ42x1 + BRICHOS
Aβ42x2
Aβ42x2 + BRICHOS

Fig. S1

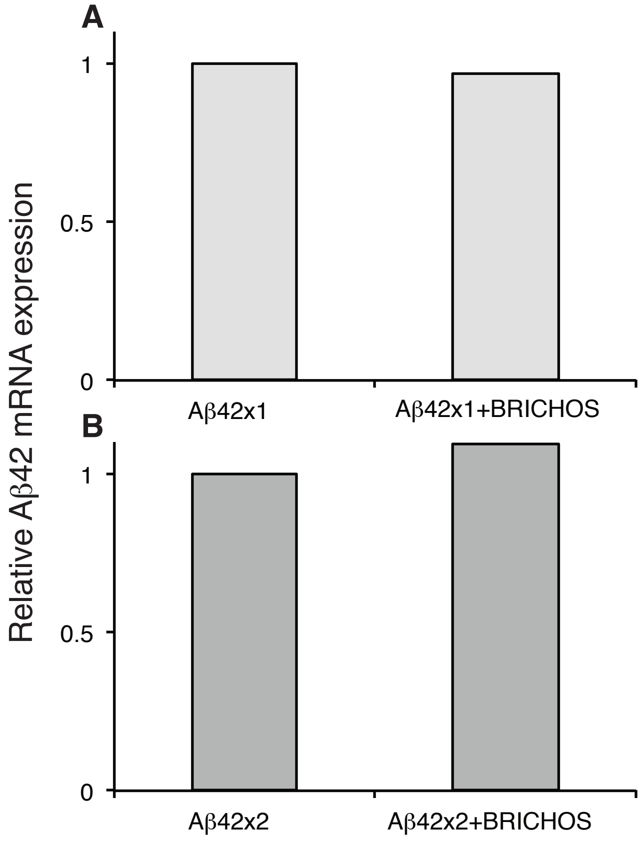
A
B
Relative Aβ42 mRNA expression
1
0.5
0
Aβ42x1
Aβ42x1+BRICHOS
Aβ42x2
Aβ42x2+BRICHOS

Fig. S2

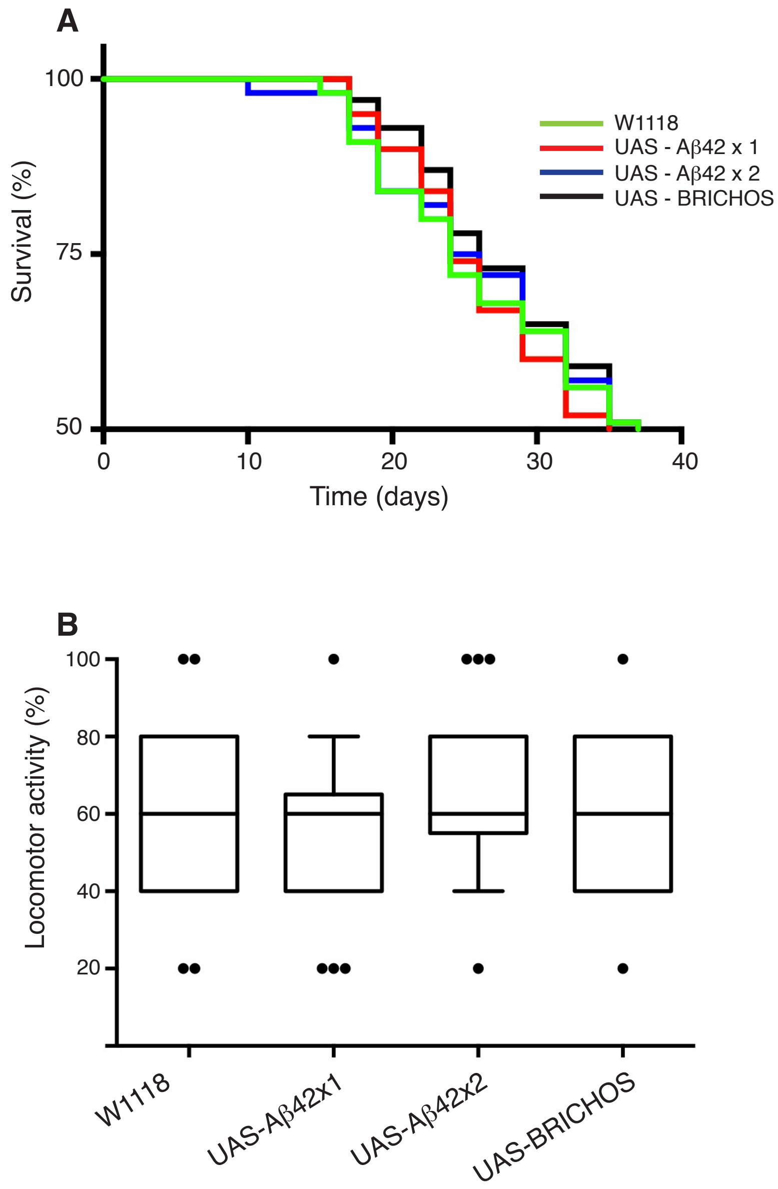
A
Survival (%)
100
75
50
0
10
20
30
40
Time (days)
W1118
UAS - Aβ42 x 1
UAS - Aβ42 x 2
UAS - BRICHOS
B
Locomotor activity (%)
100
80
60
40
20
W1118
UAS-Aβ42x1
UAS-Aβ42x2
UAS-BRICHOS

Fig. S3

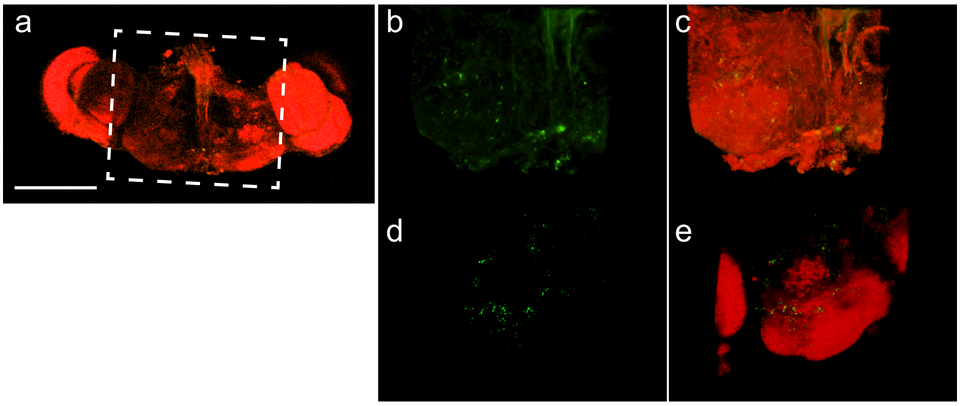

Fig. S4
